# First Case of Lateral Oropharyngectomy Performed Using the Vitom Eagle: A Novel Exoscopic System for Transoral Surgery

**DOI:** 10.1002/hed.28181

**Published:** 2025-05-05

**Authors:** Filippo Marchi, Marta Filauro, Elisa Bellini, Andrea Iandelli, Giorgio Peretti

**Affiliations:** ^1^ Unit of Otorhinolaryngology‐Head and Neck Surgery IRCCS Ospedale Policlinico San Martino Genoa Italy; ^2^ Department of Surgical Sciences and Integrated Diagnostics (DISC) University of Genoa Genoa Italy

**Keywords:** exoscope, HPV, oropharyngeal cancer, robotic surgery, VITOM 3D, VITOM EAGLE

## Abstract

**Background:**

The Vitom Eagle is a novel exoscopic system, specifically designed for transoral surgery (e.g., lateral oropharyngectomy). No prior study has compared it to existing surgical systems.

**Methods:**

This case report examines a patient affected by HPV‐related oropharyngeal squamous cell carcinoma treated with transoral lateral oropharyngectomy using the Vitom Eagle system, detailing the surgical setup, advantages, and functional and surgical outcomes.

**Results:**

The procedure achieved complete tumor resection with negative margins. Postoperative recovery was uneventful, with no complications, and functional outcomes were preserved.

**Conclusions:**

The Vitom Eagle offers significant advantages in visualization, overhead costs, and ergonomics, making it a valuable alternative tool for transoral surgeries.

## 1 Introduction

The management of oropharyngeal squamous cell carcinoma (OPSCC) has evolved significantly, driven by the increasing prevalence of HPV‐related OPSCC, which has a better prognosis than HPV‐negative cases [1]. This has spurred interest in treatment de‐intensification and minimally invasive surgical strategies, with single‐modality approaches favored for early‐stage disease [2].

Historically, OPSCC surgery involved invasive techniques like labiomandibulotomy, resulting in significant morbidity and long‐term complications [3]. Meanwhile, chemoradiotherapy or exclusive radiotherapy, while achieving similar oncological outcomes, may lead to lifelong debilitating sequelae, especially in young, healthy HPV‐positive OPSCC patients [4].

Over time, the surgical approach has evolved considerably since it was first proposed in 1951 [5]. Huet et al. proposed an approach that relies on the superior constrictor muscle as the deep surgical margin. By understanding the relationship of the deep aspect of this muscle layer, they suggested that surgery could be performed without injury to the internal carotid artery. This foundational concept paved the way for minimally invasive transoral techniques in the surgical management of OPSCC.

Innovations like robotic systems for transoral surgery (TORS) [6, 7], alongside transoral 3D exoscopic oropharyngeal surgery (TOEOS) [8, 9, 10], have transformed the field. Exoscopic systems with HD cameras and 3D visualization enable stereoscopic imaging for the entire surgical team, enhancing collaboration and broadening applications in various surgical disciplines, including head and neck, reconstructive surgery, and skull base surgery [8, 11, 12].

The Vitom Eagle (Karl Storz—Tuttlingen, Germany) represents a next‐generation exoscope, the first tailored for transoral surgeries, offering 4 K resolution, 6× optical zoom, 2× digital zoom, seamless focus adjustment, 90° field of view, and ergonomic enhancements. It supports robotic cruise technology for precise, adaptable control.

This case series is the first to report the application of the Vitom Eagle in lateral oropharyngectomy for OPSCC.

## 2 Case Report

**FIGURE 1:**
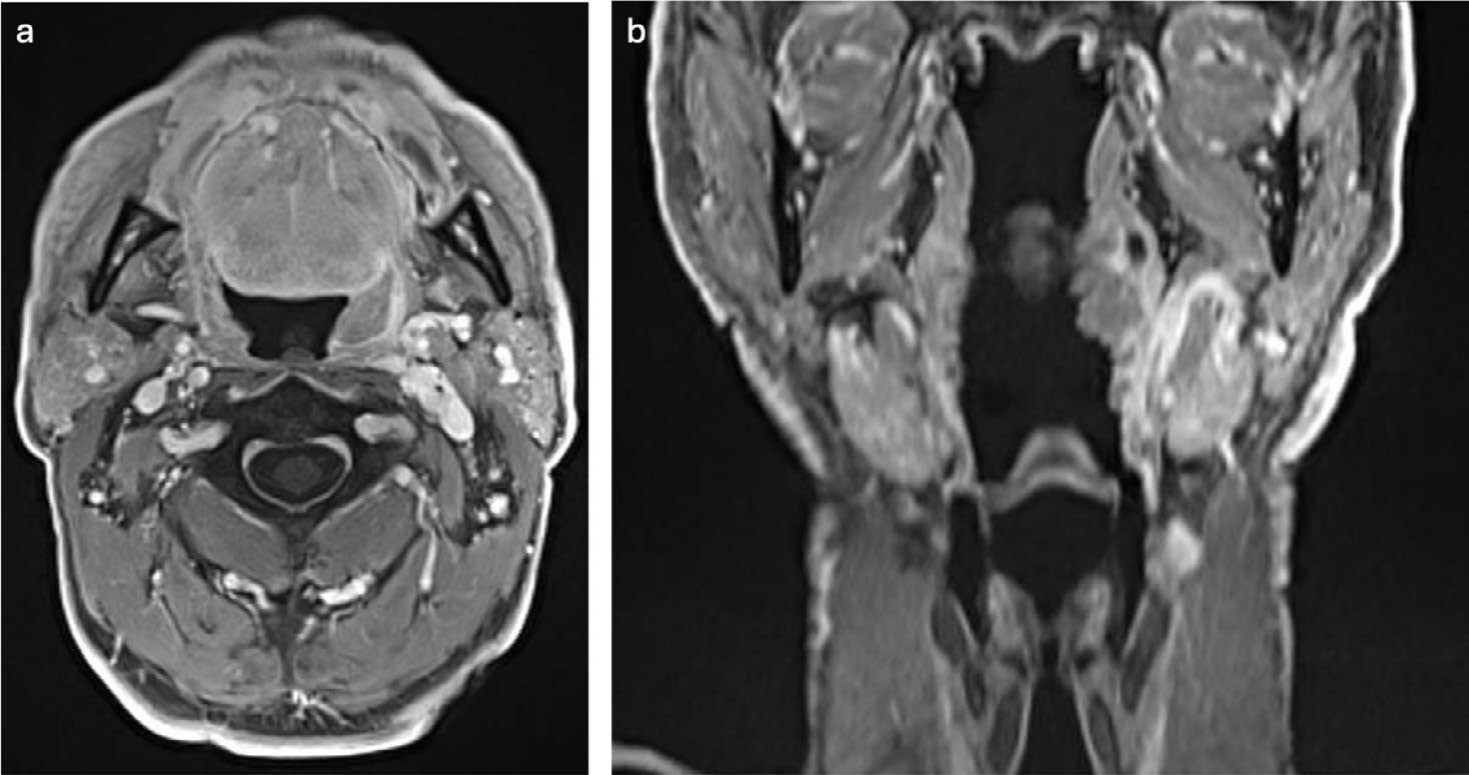
Preoperative MRI scan. (a: axial section; b: coronal section).

**FIGURE 2:**
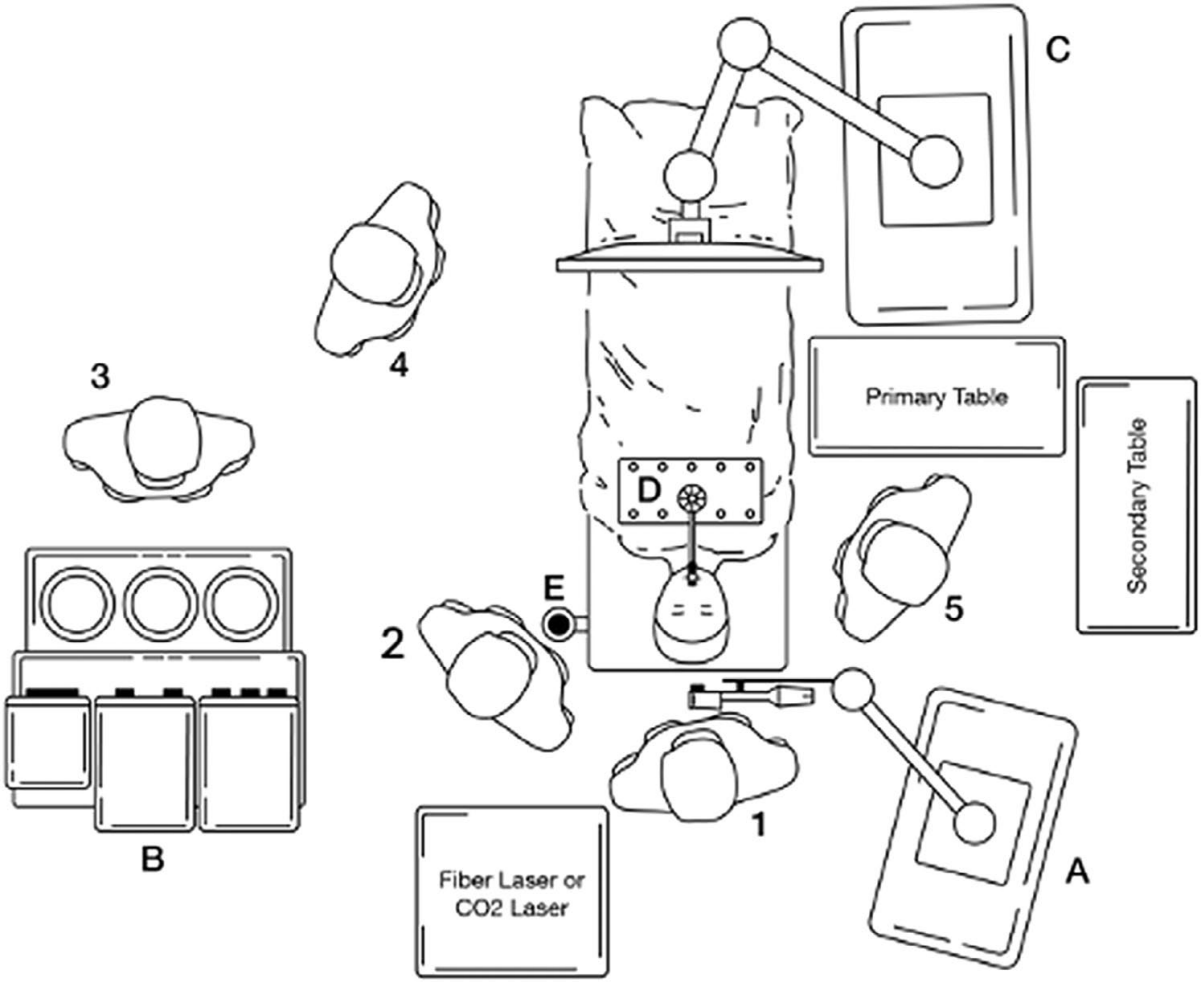
Schematic representation of TOEOS setup. A—VITOM Eagle exoscope equipped with the ARTip robotic cruise system; B—anesthesiology machine; C—4kHD‐3D monitor; D—exposure system; E—*IMAGE 1–Pilot*; 1–main surgeon; 2–assistant surgeon; 3–anesthesiology; 4–OR staff; 5–surgical nurse.

**FIGURE 3:**
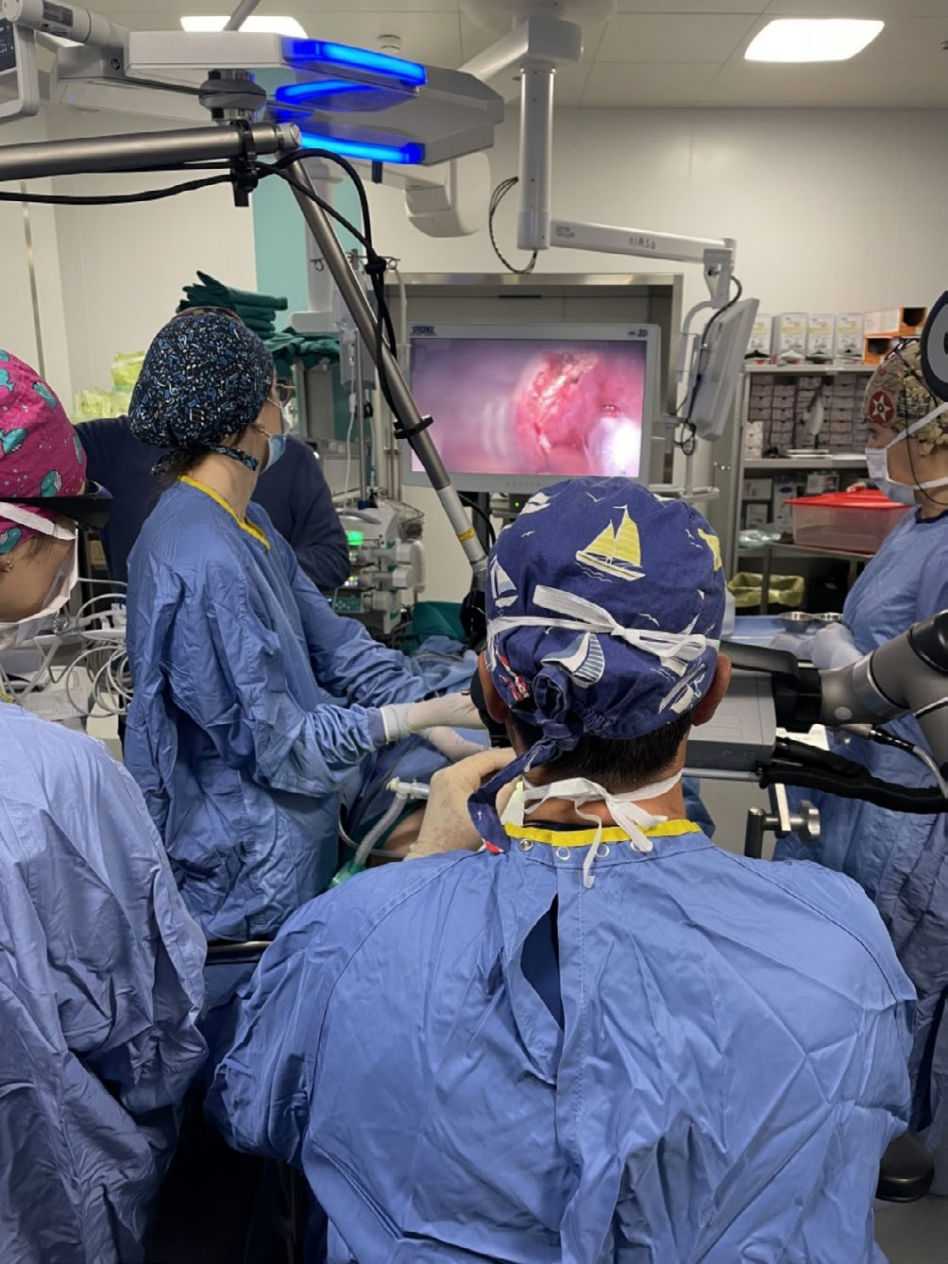
Surgeon's perspective during surgery.

A 69‐year‐old man, diagnosed with HPV‐related OPSCC of the left palatine tonsil (cT2N1) [13], underwent surgical treatment. The preoperative Magnetic Resonance Imaging (MRI) is shown in Figure 1. The Feyh‐Kastenbauer Weinstein‐O'Malley (FK‐WO) retractor provided adequate exposure of the entire oropharynx. A type 2 lateral oropharyngectomy combined with a selective ipsilateral neck dissection (SND) of IIa‐IIb‐III‐IV levels was performed [14]. The surgical setup closely resembled that of TORS, with one assistant surgeon positioned at the side of the patient's head to manage additional instruments, while another adjusted the image framing and focus using the IMAGE 1 Pilot system and foot pedal. This ensured optimal visualization and procedural efficiency (Figure 2). Figure 3 presents the surgeon's perspective throughout the entire procedure. The final histopathological examination revealed a maximum tumor diameter of 28 mm, evidence of lymphovascular invasion, no perineural invasion, and clear surgical margins. One nodal metastasis at level IIa was found on the left side, measuring 35 mm at its largest dimension. The final classification according to AJCC staging system was pT2 N1 HPV‐related OPSCC. The mucosal and deep margins were confirmed to be free of tumor, the closest margin was the inferior (2 mm). During the initial postoperative period, the patient received nutrition via parenteral feeding. Speech therapy rehabilitation was initiated on the fifth postoperative day, progressing to a soft diet and ultimately transitioning to a full oral diet by the time of discharge. As part of the surgical procedure, a temporary tracheotomy was performed as a precautionary measure to mitigate the risk of postoperative bleeding. The tracheotomy cannula was removed on postoperative day 8. Since this was the first case treated with this novel tool, we preferred to perform a temporary tracheotomy for safety reasons. The patient's total hospitalization lasted 15 days.

## 3 Discussion

The Vitom Eagle represents a major advancement in exoscopic technology, offering distinct advantages over the Vitom 3D and da Vinci Xi, particularly for transoral procedures like lateral oropharyngectomy (Table 1). Its design addresses critical surgical needs by providing exceptional visualization and versatility at every stage of the procedure. The overall cost of the system is roughly one‐tenth the cost of the robotic systems.

**TABLE 1:** Comparison of characteristics between Vitom 3D, Vitom Eagle, and Da Vinci Xi.

Features	Transoral exoscopic surgery		Transoral robotic surgery	
	Vitom 3D	Vitom Eagle	Da Vinci Xi	SP system
Energy device	CO_2_ laser, fiber lasers, monopolar, bipolar, ultrasound	CO_2_ laser, fiber lasers, monopolar, bipolar, ultrasound	Monopolar, bipolar	Monopolar, bipolar, lumenis attachment for Co_2_ laser
Instruments	Rigid straight or curved instruments	Rigid straight or curved instruments	Jointed arms with 3D range of motion	Jointed arms with 3D range of motion
Illumination	No spots	Yes	Yes	Yes
Surgical view	Straight 0°	Straight 0°	Straight or angled 0° to 30°	Flexible HD Camera
Feedback	Tactile and visual	Tactile and visual	Visual	Visual
Exposure	Compatible with any mouth opener and laryngoscope	Compatible with any mouth opener and laryngoscope	Compatible with any mouth openers	Compatible with any mouth gag/opener
Target(s)	Oropharynx, Supraglottis, Glottis, Cervical esophagus	Oropharynx, Supraglottis, Glottis, Cervical esophagus	Oropharynx, Supraglottis	Oropharynx, Supraglottis, Limited Glottic/hypopharyngeal
Assistants	3d‐vision available for all the OR staff	3d‐vision available for all the OR staff	3d‐vision available for the first surgeon	3d‐vision available for the first surgeon
Costs	**≈**150.000 $	**≈**200.000 $	**≈**2 million $	**≈**2 million $
Disposable	No	No	Yes	Yes
Zoom	4 × Digital	6× Optical + 2 × Digital	10× Digital	4 × Digital
Resolution	Full HD	4 K	Full HD	Full HD

At the same time, recent studies have highlighted the significant benefits of the da Vinci Single‐Port (SP) system, particularly when compared to multiport systems [15, 16].

From a technical perspective, the SP system offers improved access, visualization, and maneuverability due to its flexible, single‐arm design. Additionally, it provides greater proficiency with the incorporation of a third surgical arm, making it particularly useful for procedures in deep anatomical spaces, such as the oropharynx and larynx [17].

From a clinical perspective, de Virgilio et al. demonstrated that the SP system facilitates precise tumor resection, enhances preservation of surrounding tissues, and reduces morbidity [18].

Nevertheless, the SP system still has limitations in glottic surgery due to the use of coarser instruments, unlike the phonosurgical instruments that can be utilized with the Vitom Eagle.

Here We Provide a Step‐By‐Step Description of How Each Technical Aspect Overcomes the Drawbacks of Previous Robotic and Exoscopic Systems.

**FIGURE 4:**
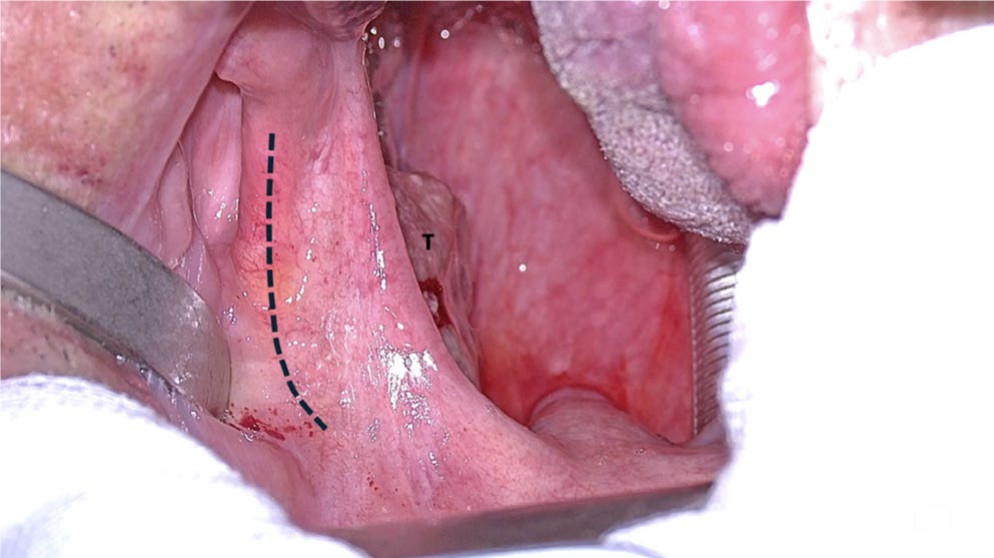
Intraoperative view of the left oropharynx (dotted line: incision line along the pterygomandibular raphe; T: tumor).

*Step 1: Mucosal Incisions*: In this clinical case we present, the mucosal incision was performed using a monopolar device, but the Vitom Eagle offers the possibility of using its stable CO_2_ laser coupling, which ensures precise tissue ablation and coagulation while minimizing mucosal damage [19] (Figure 4). This allows the mucosa of the pterygomandibular raphe to be incised with significantly less thermal injury compared to the monopolar device, promoting faster healing. Moreover, the Vitom Eagle enables tactile feedback for pterygomandibular raphe identification, a key advantage over the robotic‐only approach.

**FIGURE 5:**
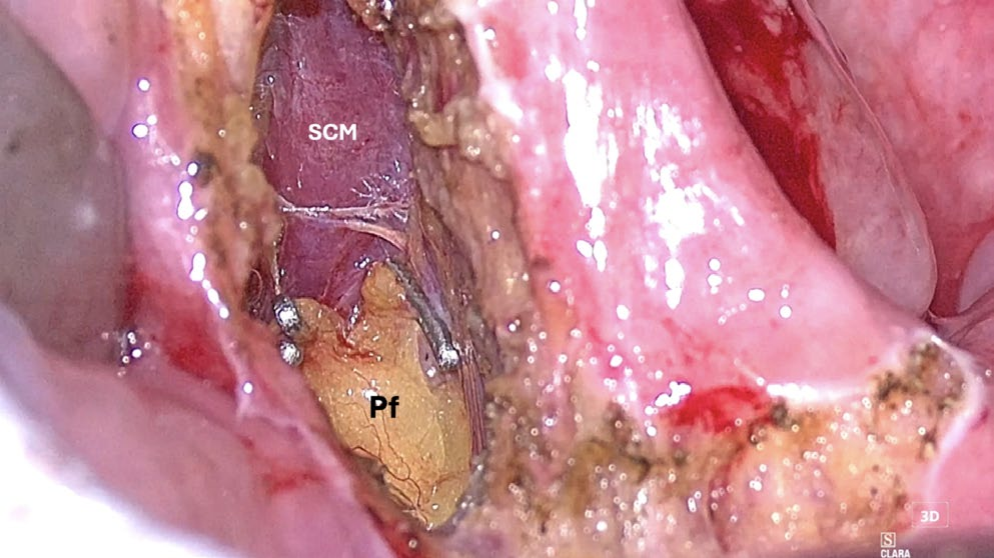
Muscle dissection. (SCM: superior constrictor muscle; Pf: parapharyngeal fat).

**FIGURE 6:**
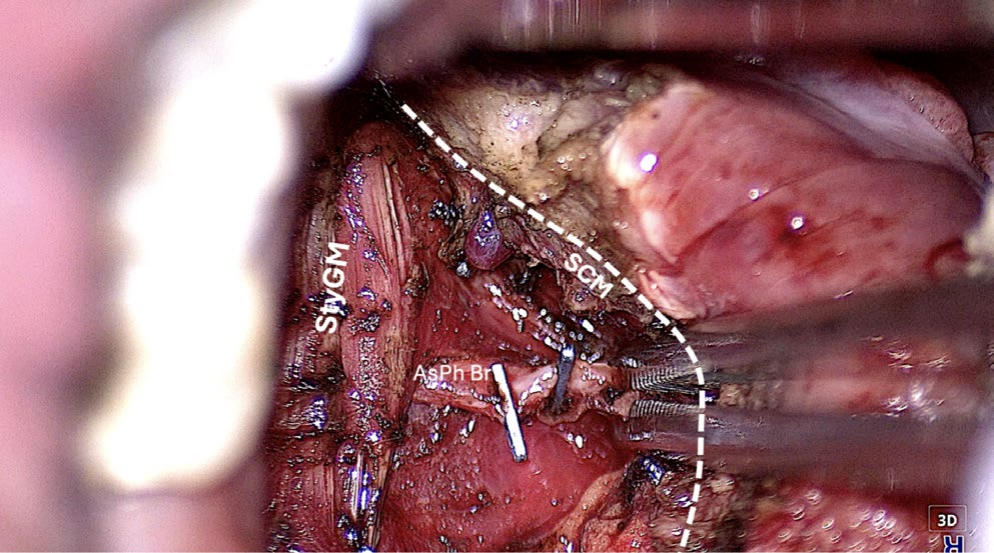
Muscle dissection (StyGM: styloglossus muscle; AsPh Br: ascending pharyngeal artery branch; dotted line SCM: superior constrictor muscle).

*Step 2: Muscle Dissection*: The Eagle's 4 K resolution delivers superior visualization compared to the HD capabilities of the Vitom 3D and TORS 3D systems. Its dual zoom (6× optical and 2× digital) provides seamless magnification, essential for narrow surgical fields like the oropharynx. This facilitates precise dissection of the superior constrictor and medial pterygoid muscles (Figure 5). Upon completion of the dissection of the superior constrictor muscle, the parapharyngeal fat and the styloglossus muscle are identified (Figure 6).

**FIGURE 7:**
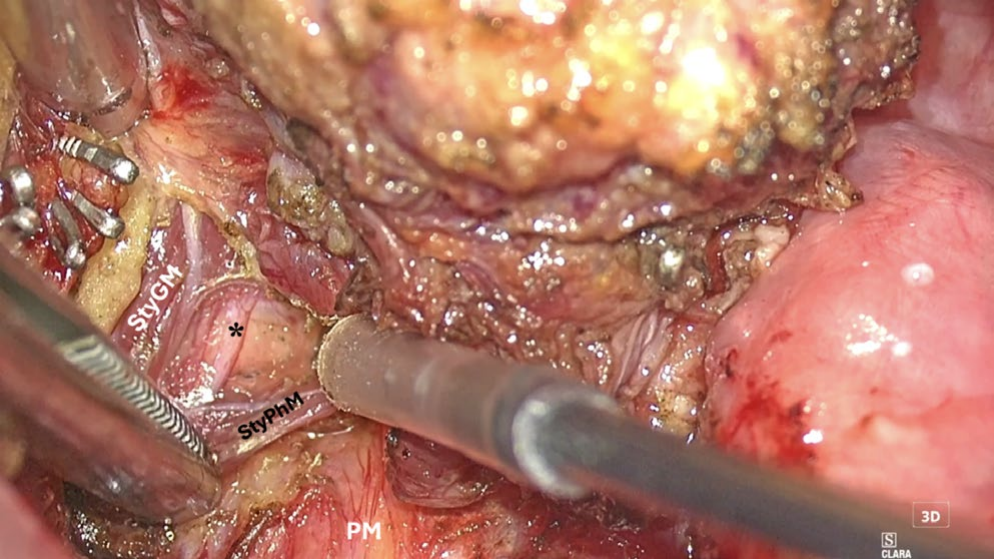
Deep plane/parapharyngeal space dissection (StyGM: styloglossus muscle; *glossopharyngeal nerve; StyPhM: stylopharyngeus muscle; PM: prevertebral muscles plane).

**FIGURE 8:**
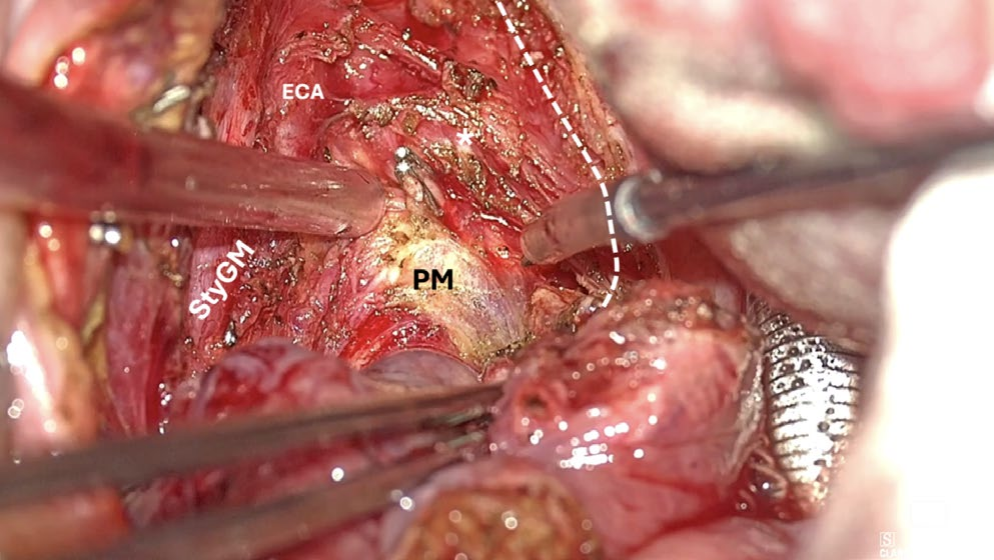
Posterior/inferior pharyngeal wall dissection (StyGM: styloglossus muscle; *stylopharyngeus muscle transected; ECA: external carotid artery; PM: prevertebral muscles plane).

*Step 3: Deep plane/parapharyngeal space dissection*: The Vitom Eagle facilitates a four‐hands procedure, allowing the assistant to use two additional instruments for tissue manipulation (Figure 7). This enables the retraction of the fat pad and the eventual transection of the bellies of the stylopharyngeus and styloglossus muscles while simultaneously protecting the carotid artery (Figure 8). The outstanding optical magnification, coupled with 4 K resolution, allows for the identification of small anatomical structures, such as the pharyngeal branch of the glossopharyngeal nerve (Figure 7). Moreover, the tactile feedback provides prompt identification of the styloid process, a crucial landmark in parapharyngeal surgery.

**FIGURE 9:**
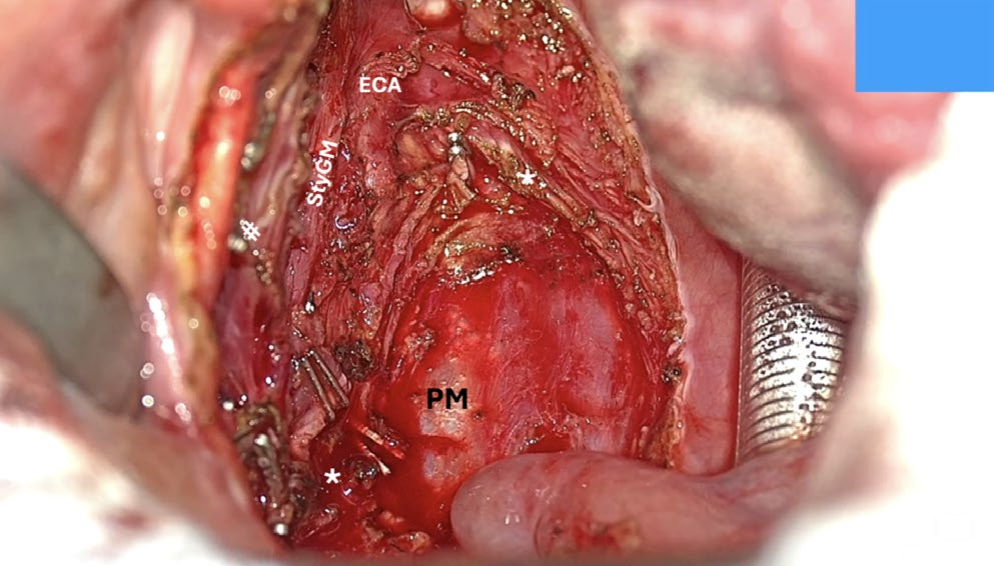
Tumor bed at the end of resection (StyGM: styloglossus muscle; *stylopharyngeus muscle transected; PM: prevertebral muscles plane; ECA: external carotid artery; ^#^medial pterygoid muscle).

*Step 4: Posterior/Inferior Pharyngeal Wall Dissection*: In the later stages, the Eagle's ergonomic features significantly enhance workflow efficiency. Once a substantial portion of the surgical specimen is delivered, the dissection continues along the posterior wall, detaching the origin of the constrictor from the prevertebral muscles' fascia, cutting the pharyngeal insertion of the stylopharyngeus muscle, and proceeding inferiorly toward the tongue base and the glossotonsillar sulcus. In the infero‐anterior and posterior portions of the dissection, where the surgical field is deeper, the benefits of the Vitom Eagle become particularly evident. Its stepless focus mechanism and intuitive controls, operated via the IMAGE1 PILOT system and foot pedals, minimize interruptions and reduce surgeon fatigue, particularly during lengthy procedures. The illumination, aligned along the same optical axis as the observation, effectively minimizes shadowing (Figure 8). Furthermore, the reduced diameter of the light beam spot allows for significantly better lighting in narrow cavities and access points. Its advanced magnification and focus capabilities, even under low‐light or limited‐visibility conditions, streamline challenging steps such as posterior‐inferior wall incision and dissection (Figure 9).

While the Vitom Eagle exoscope offers advantages in ergonomics and workflow efficiency, the da Vinci SP system remains a leading robotic‐assisted option for minimally invasive transoral procedures, particularly where deep access and enhanced articulation are required. Moreover, it accommodates reusable tools like CO_2_ laser and diode laser, reducing operational costs and dependency on proprietary systems (Table 1). Additionally, it supports forceps and tools suitable for transoral laryngeal surgery and thermal or ultrasonic instruments that can also be utilized in open neck procedures, further enhancing its versatility across different surgical applications [9, 10]. This flexibility allows for broader use while significantly lowering the costs associated with disposable robotic instruments [20](Table 1). In addition to the advantages described in the reported case, one of the most significant benefits of the exoscope is its versatility in addressing other anatomical subsites, such as the supraglottic larynx (similar to TORS). However, unlike TORS, the exoscope can more easily be used in the glottis and cervical esophagus as it accommodates finer instruments (Table 1).

In the case report, the Vitom Eagle achieved proper surgical outcomes, including complete tumor resection with negative margins and preserved swallowing and phonatory functions. In a previous study, we demonstrated that its predecessor, the Vitom 3D, exhibits post‐treatment quality of life comparable to that of patients treated with other strategies [7].

As reported by Piazza et al. transoral exoscopic surgery projects the surgical field on a high‐definition screen visible to the entire team, fostering collaboration and minimizing strain during long procedures [21].

With 3D 4 K high‐definition visualization, enhanced precision, the Vitom Eagle is an appealing alternative for transoral surgery. As robotic‐assisted techniques advance, exoscopic and robotic approaches may serve complementary roles depending on surgical complexity, anatomical site, and institutional resources.

Purpose‐built for transoral procedures, the Vitom Eagle sets a new standard in precision and efficiency for narrow surgical fields. Its integration of fluorescence imaging, laser technology, and conventional transoral instruments, along with great workflow efficiency, makes it a versatile alternative to optical microscopes, robotic systems, and earlier exoscopes.

The main limitation we encountered with the Vitom Eagle and other existing exoscopes is performing procedures in anatomical areas where the mucosal surface is coaxial to the visual axis. An example is the lower portion of the tongue base toward the vallecula, where the cutting angle may be blind in certain unfavorable anatomical conditions, especially when dual‐angled instruments are unavailable.

While the Vitom Eagle offers significant advantages in visualization and cost‐effectiveness, further comparative studies—particularly with the da Vinci SP system—are needed to fully assess its role relative to established transoral approaches.

## Author Contributions

F.M., M.F., G.P.: study conception; F.M., E.B.: study design; F.M., M.F., A.I.: data collection; F.M., A.I., E.B.: draft manuscript preparation; G.P. approved the final version of the manuscript.

## Ethics Statement

This study was approved by the Institutional Ethics Committee. (CER Liguria: 220/2024). The research was conducted ethically, with all study procedures being performed in accordance with the requirements of the World Medical Association's Declaration of Helsinki. Written informed consent was obtained from each participant/patient for study participation and data publication.

## Conflicts of Interest

The authors declare no conflicts of interest.

## Data Availability

The data that support the findings of this study are available from the corresponding author upon reasonable request.
